# Joint sparse reconstruction of multi-contrast MRI images with graph based redundant wavelet transform

**DOI:** 10.1186/s12880-018-0251-y

**Published:** 2018-05-03

**Authors:** Zongying Lai, Xinlin Zhang, Di Guo, Xiaofeng Du, Yonggui Yang, Gang Guo, Zhong Chen, Xiaobo Qu

**Affiliations:** 1 Department of Electronic Science, Fujian Provincial Key Laboratory of Plasma and Magnetic Resonance, Xiamen University, Xiamen, 361005 China; 2 Department of Communication Engineering, Xiamen University, Xiamen, 361005 China; 3 School of Computer and Information Engineering, Fujian Provincial University Key Laboratory of Internet of Things Application Technology, Xiamen University of Technology, Xiamen, 361024 China; 4 Department of Radiology, No.2 Hospital Xiamen, Xiamen, 361021 China

**Keywords:** Magnetic resonance imaging, Fast imaging, Image reconstruction, Sparsity

## Abstract

**Background:**

Multi-contrast images in magnetic resonance imaging (MRI) provide abundant contrast information reflecting the characteristics of the internal tissues of human bodies, and thus have been widely utilized in clinical diagnosis. However, long acquisition time limits the application of multi-contrast MRI. One efficient way to accelerate data acquisition is to under-sample the k-space data and then reconstruct images with sparsity constraint. However, images are compromised at high acceleration factor if images are reconstructed individually. We aim to improve the images with a jointly sparse reconstruction and Graph-based redundant wavelet transform (GBRWT).

**Methods:**

First, a sparsifying transform, GBRWT, is trained to reflect the similarity of tissue structures in multi-contrast images. Second, joint multi-contrast image reconstruction is formulated as a ℓ_2, 1_ norm optimization problem under GBRWT representations. Third, the optimization problem is numerically solved using a derived alternating direction method.

**Results:**

Experimental results in synthetic and in vivo MRI data demonstrate that the proposed joint reconstruction method can achieve lower reconstruction errors and better preserve image structures than the compared joint reconstruction methods. Besides, the proposed method outperforms single image reconstruction with joint sparsity constraint of multi-contrast images.

**Conclusions:**

The proposed method explores the joint sparsity of multi-contrast MRI images under graph-based redundant wavelet transform and realizes joint sparse reconstruction of multi-contrast images. Experiment demonstrate that the proposed method outperforms the compared joint reconstruction methods as well as individual reconstructions. With this high quality image reconstruction method, it is possible to achieve the high acceleration factors by exploring the complementary information provided by multi-contrast MRI.

## Background

Multi-contrast images in magnetic resonance imaging (MRI) provide abundant contrast information reflecting the characteristics of the internal tissues of human bodies, and thus have been utilized in clinical diagnosis. However, long acquisition time limits the application of multi-contrast MR imaging.

Under-sampling the k-space data and reconstructing images with sparsity constraint is one efficient way to accelerate MRI sampling [1–5]. However, the data acquisition factor is limited since images are compromised when images are reconstructed individually. The previous work [6] suggested to use another fully-sampled contrast image to train an adaptive sparse representation with Graph-based redundant wavelet transform (GBRWT) and then greatly improve the reconstructed images [7]. This approach, however, cannot reduce the overall acceleration factor in data acquisition because of the full sampling in another contrast images [6]. Thus, to further accelerate multi-contrast MRI, under-sampling all multi-contrast images, e.g.T1 weighted (T1W), T2 weighted (T2W) and proton density weighted (PDW) MRI images, and maintain high quality image reconstruction are expected.

The MRI image structures under different contrast settings are the same due to the multiple acquisitions of the same anatomical cross section [6, 8–12]. Thus, non-zero coefficients may occur at the same spatial locations in the sparsifying transform domains, e.g. finite difference, wavelet transform [2] and patch-based sparse transformations [13–16]. Therefore, it is possible to improve the image reconstruction if this extra information is incorporated into sparse image reconstruction [17].

Sparse representation capability plays a key role in sparse MRI reconstruction. The GBRWT [6, 7] transform was verified to have good sparsification capability for MRI images. The main step of GBRWT transform is to construct a graph to find new permutations adaptive to target image structures, and then to obtain the sparser transformation with wavelet filters acting on the permutated smooth signals. However, if high acceleration factor is set, very limited information will be provided for single image thus the reconstruction will be compromised. Thus, the combining merits of joint reconstruction and GBRWT are expected.

In this study, we propose to reconstruct the multi-contrast MRI with adaptive GBRWT sparse representations and joint sparsity among multi-contrast images. An alternating direction method with continuity (ADMC) [18] algorithm is introduced to solve the joint ℓ_2, 1_-norm minimization problem. The proposed approach will be compared with the joint sparse reconstruction method based on shift invariant discrete wavelet transform (SIDWT) [17] and Bayesian compressed sensing (BCS) [19].

## Methods

The under-sampled k-space data of multi-contrast MRI images are expressed as

1 $$ \mathbf{y}=\mathbf{UFx}+\boldsymbol{\upvarepsilon}, $$

where **x** = [**x**_1_; ⋯; **x**_*T*_] denotes the column stacked multi-contrast images, *T* the number of contrasts. **y** = [**y**_**1**_; ⋯; **y**_*T*_] the column stacked under-sampled k-space data, **ε** noises in the sampled data. The **UF** is the under-sampling operator in the Fourier space, which can be expressed as

2 $$ \mathbf{UF}=\left[\begin{array}{ccc}{\mathbf{U}}_1{\mathbf{F}}_1& 0& 0\\ {}0& \ddots & 0\\ {}0& 0& {\mathbf{U}}_T{\mathbf{F}}_T\end{array}\right]. $$

Each **U**_*i*_**F**_*i*_, (*i* = 1, ⋯, *T*) acts on one of the multi-contrast images. We adopt different sampling patterns, i.e. **U**_1_ ≠ ⋯ ≠ **U**_*i*_ ≠ ⋯ ≠ **U**_*T*_, and the same Fourier transform bases, i.e. **F**_1_ = ⋯ = **F**_*i*_ = ⋯ = **F**_*T*_ for each image of individual contrast.

**Fig. 1:**
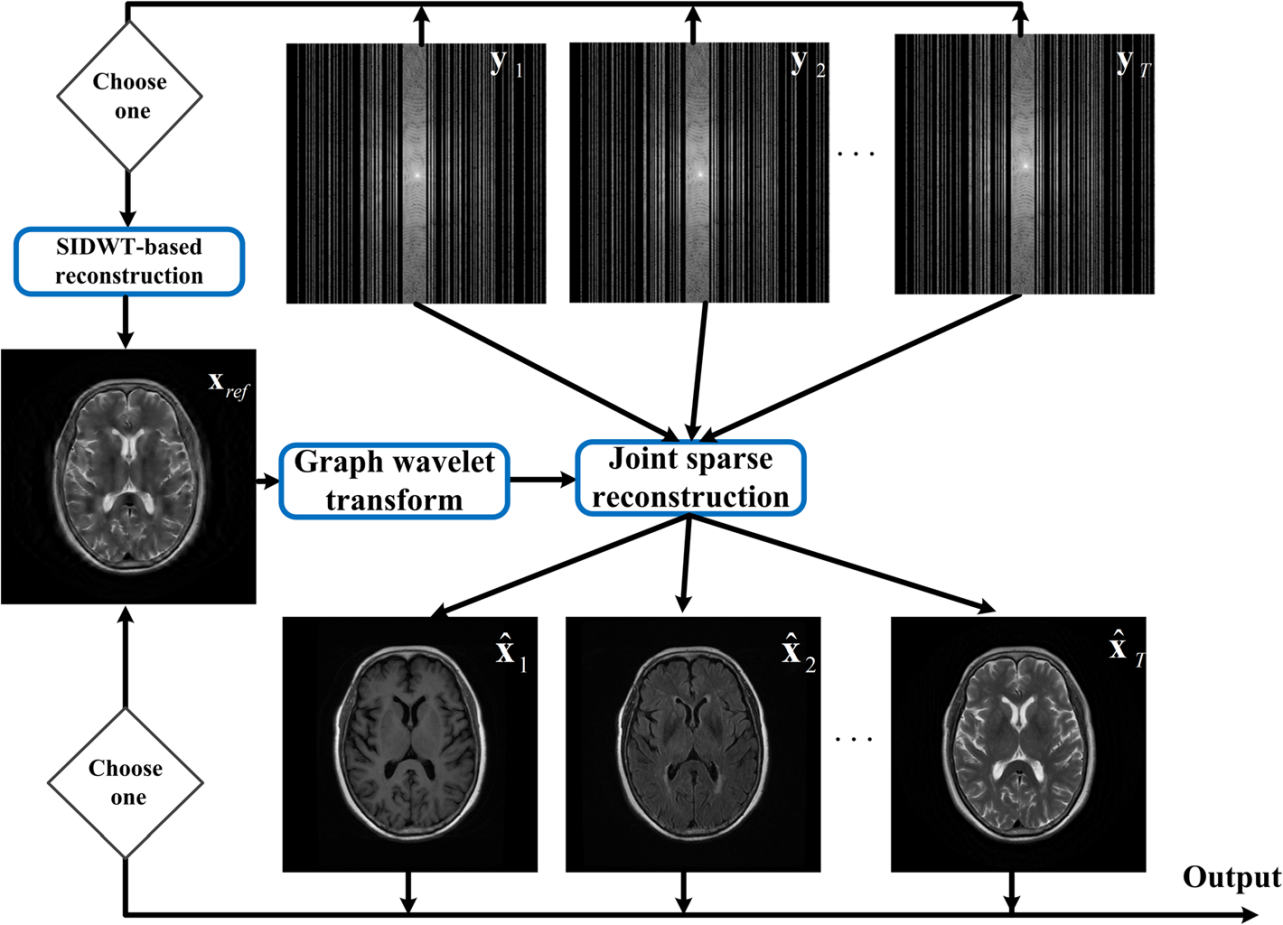
Flowchart of the proposed method. The **x**_*ref*_ denotes the reference image used to train the graph wavelet transform, **y**_1_, **y**_2_, ⋯, **y**_*T*_ denote the under-sampled k-space data of multi-contrast images, ${\widehat{\mathbf{x}}}_1,{\widehat{\mathbf{x}}}_2,\cdots, {\widehat{\mathbf{x}}}_T$ denote the reconstructed images

The flowchart of the proposed joint sparse reconstruction is shown in Fig. 1. Reconstructed image based on SIDWT [17] is adopted as reference image to train the GBRWT from the under-sampled k-space data, because SIDWT can mitigate the blocky artifacts introduced by orthogonal wavelet transform and better preserve the structures in the target images [15, 16]. With GBRWT as the sparse representation, multi-contrast images can be simultaneously reconstructed by implementing joint sparsity constraints on these transformation coefficients.

### Graph-based redundant wavelet transform

Given a reference image, the GBRWT is achieved by carrying out redundant wavelet transform on permuted signals of new orders [7]. The new orders are found in weighted graph constructed from the reference image, in which image patches collected by a sliding window serve as the vertex and the patch similarities computed using *w*_*m*, *n*_ = *w*(**b**_*m*_, **b**_*n*_) = ‖**b**_*m*_ − **b**_*n*_‖_2_ (where **b**_*m*_ and **b**_*n*_ denote the *m*^*th*^ and the *n*^*th*^ patches) serve as the weight. The new orders are obtained by finding the shortest possible path on the patch-based graph [7, 20]. Then, redundant wavelet transform is performed on permuted pixels to achieve sparse representation.

**Fig. 2:**
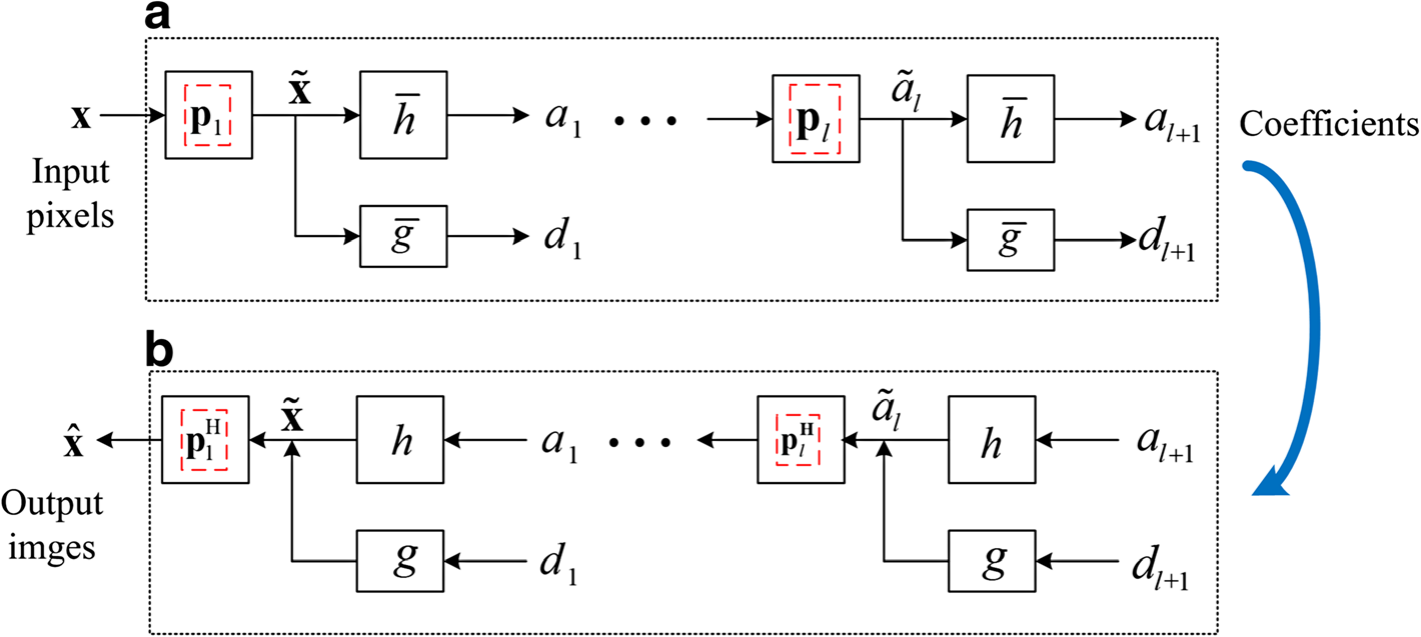
The permutation and wavelet filtering on re-ordered image pixels, **a** and **b** are the forward and inverse transforms

The process of permutation and wavelet filtering in GBRWT is shown in Fig. 2. In the *l*^*th*^ level decomposition, the input signal *a*_*l*_ will be first reordered by permutation matrix **P**_*l*_, whose inverse process is ${\mathbf{P}}_l^{\mathrm{H}}$ and satisfied ${\mathbf{P}}_l^{\mathrm{H}}{\mathbf{P}}_l=\mathbf{I}$. Then, non-decimated wavelet transformation **Φ**_*l*_, whose inverse process is ${\boldsymbol{\Phi}}_l^{\mathrm{H}}$ and satisfied ${\boldsymbol{\Phi}}_l^{\mathrm{H}}{\boldsymbol{\Phi}}_l=\mathbf{I}$, are performed on the re-ordered pixels. The output *a*_*l* + 1_ and *d*_*l* + 1_ of *l*^*th*^ level non-decimated decomposition will be of the same size with the input signal ${\tilde{a}}_l$. Let **Φ**_*l*_**P**_*l*_ be the *l*^*th*^ level decomposition of GBRWT, and ${\mathbf{P}}_l^{\mathrm{H}}{\boldsymbol{\Phi}}_l^{\mathrm{H}}$ be corresponding composition process, and then **Φ**_*l*_**P**_*l*_ satisfies the following property

3 $$ {\mathbf{P}}_l^{\mathrm{H}}{\boldsymbol{\Phi}}_l^{\mathrm{H}}{\boldsymbol{\Phi}}_l{\mathbf{P}}_l=c\mathbf{I}, $$

where *c* denotes the redundancy of GBRWT transform. It has been verified that GBRWT provides sparser representations than traditional wavelet transform, thus can improve the MRI image reconstruction [7].

### Joint sparsity of multi-contrast image coefficients

**Fig. 3:**
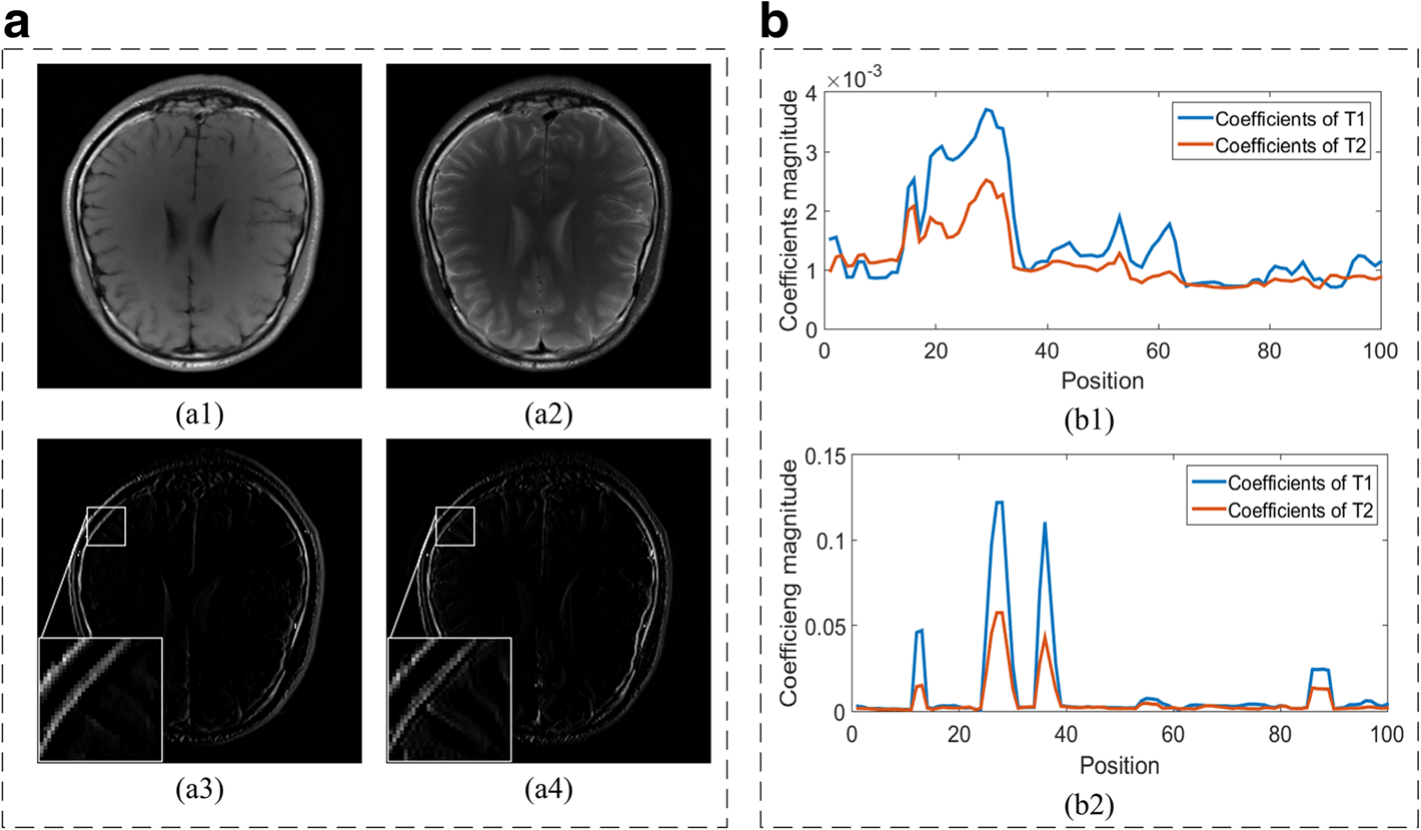
Joint sparsity property of multi-contrast image coefficients. Group sparsity for traditional wavelet transform is shown in (A), (a1-a4) are images of two contrasts (T1W and T2W MRI images) and their corresponding coefficients in the 3rd sub-bands of traditional redundant Haar wavelet transform. Group sparsity of GBRWT is shown in (B) and (b1-b2) are two selected fragments of GBRWT coefficients

Multi-contrast MRI images are obtained by different parameter settings, but share the same anatomical cross section [6–8]. The image structures corresponding to tissue locations remain unchanged with contrast varied, which leads to spatial position-related coefficients. Joint sparsity means that, under appropriate sparsifying transforms, the positions of non-zero coefficients correspond directly to same spatial locations in multiple images. Figure 3 shows that the non-zero transform coefficients of two contrast images occur at the same positions in the Haar wavelets transform and GBRWT domains. Thus, the joint sparsity of GBRWT provides extra information on images and may further improve the reconstruction of multi-contrast images.

### Problem formulation

**Fig. 4:**
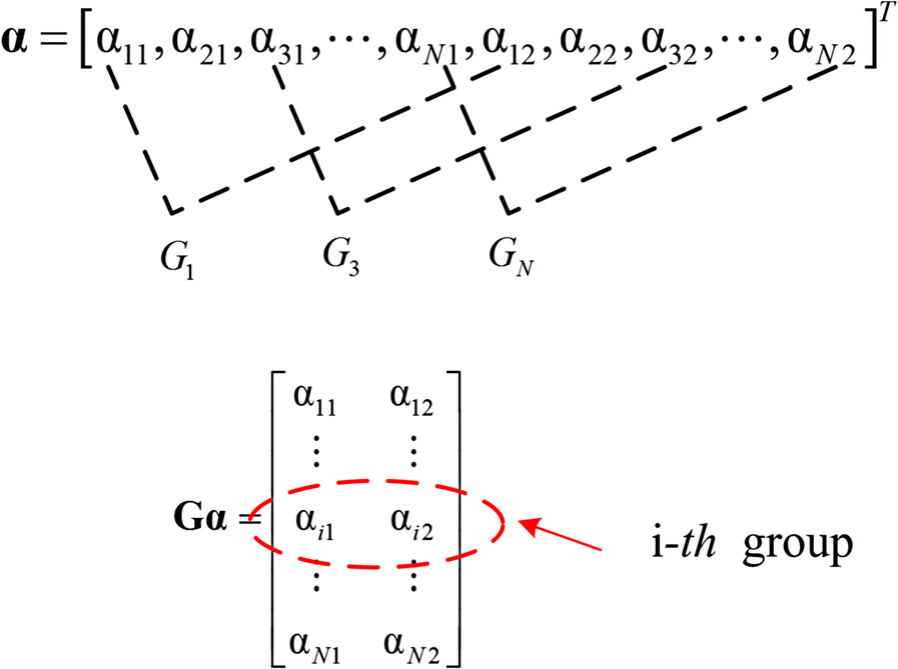
The grouping operator **G** and the grouped GBRWT coefficients

The joint sparsity promoting problem in multi-contrast MRI image reconstruction with GBRWT is solved using the mixed ℓ_2, 1_ norm minimization [9, 21, 22]:

4 $$ \underset{\mathbf{x}}{\min }{\left\Vert \mathbf{G}\boldsymbol{\Psi } \mathbf{x}\right\Vert}_{2,1}\kern0.75em s.t.\kern0.5em {\left\Vert \mathbf{UFx}-\mathbf{y}\right\Vert}_2^2\le {\sigma}^2, $$

where, $$ \boldsymbol{\Psi} =\left[\begin{array}{ccc}{\boldsymbol{\Psi}}_g& 0& 0\\ {}0& \ddots & 0\\ {}0& 0& {\boldsymbol{\Psi}}_g\end{array}\right] $$ and **Ψ**_g_ denotes the GBRWT representation, in which *l*^*th*^ level decomposition be expressed as **Φ**_*l*_**P**_*l*_. Let **α** = **Ψx** be the corresponding coefficients, then for an image set which includes *T* kinds of contrasts, the column stacked coefficients can be expressed as: **α** = [**α**_1_; ⋯; **α**_*T*_]. The role of grouping operator **G** is to reshape the column stacked coefficients of multi-contrast MRI images into a matrix as shown in Fig. 4. Then, one column of **Gα** stands for coefficients of one image, and one row forms a group.

The ℓ_2, 1_ norm is defined as

5 $$ {\left\Vert \mathbf{G}\boldsymbol{\upalpha } \right\Vert}_{2,1}=\sum \limits_{i=1}^N{\left(\sum \limits_{j=1}^T{\left|{\boldsymbol{\upalpha}}_{ij}\right|}^2\right)}^{1/2}, $$

where, **G** is the group operator, *N* is the number of transform coefficient and *T* the number of contrast.

### Numerical algorithm

The alternating direction method with continuation [18] is incorporated in the ℓ_2, 1_ norm optimization. Let **α** = **Ψx**, the objective in Eq. (4) can be rewritten as

6 $$ \underset{\mathbf{x},\boldsymbol{\upalpha}}{\min }{\left\Vert \mathbf{G}\boldsymbol{\upalpha } \right\Vert}_{2,1}{\displaystyle \begin{array}{cc}& s.t.\end{array}}\kern0.5em {\left\Vert \mathbf{UFx}-\mathbf{y}\right\Vert}_2^2\le {\sigma}^2,\boldsymbol{\upalpha} =\boldsymbol{\Psi} \mathbf{x}. $$

Furthermore, the objective function in (6) can be over-relaxed to be unconstraint as

7 $$ \underset{\mathbf{x},\boldsymbol{\upalpha}}{\min }{\left\Vert \mathbf{G}\boldsymbol{\upalpha } \right\Vert}_{2,1}+\frac{\beta }{2}\ {\left\Vert \boldsymbol{\upalpha} -\boldsymbol{\Psi} \mathbf{x}\right\Vert}_2^2+\frac{\lambda }{2}{\left\Vert \mathbf{UFx}-\mathbf{y}\right\Vert}_2^2. $$

The *λ* is a parameter to balance the sparsity and data fidelity. The *β* is fixed in the inner loops and changes continuously to achieve optimal reconstruction in the outer loops. When *β* → ∞, the solution of Eq. (7) approaches to that of Eq. (6). When *β* is fixed, **x** and **α** will be computed alternatively by the following two steps:

1. With **x** fixed, **α** will be computed by solve the objective:

8 $$ \underset{\boldsymbol{\upalpha}}{\min }{\left\Vert \mathbf{G}\boldsymbol{\upalpha } \right\Vert}_{2,1}+\frac{\beta }{2}\ {\left\Vert \boldsymbol{\upalpha} -\boldsymbol{\Psi} \mathbf{x}\right\Vert}_2^2. $$

**Table:** 

**Algorithm 1**: Joint multi-contrast MRI reconstruction based on GBRWT	
**Parameters:** *λ* **Input:**	
k-space data **y** = [**y**_1_; ⋯; **y**_*T*_]; *g* levels of permutation orders **P**_*j*_, {*j* = 1, ⋯, *g*}; regularization parameter *λ*; tolerance of inner loop *η* = 10^−4^.	
**Initialization**: **x** = (**UF**)^H^**y**, **x**_*previous*_ = **x**, *β* = 2^6^.	
**Main**:	
While *β* ≤ 2^12^	
(1) Given **x**, solving **α** by computing Eq. (10) for each group of coefficients **α**^*i*^, {*i* = 1, ⋯, *N*};	
(2) Applying **α** into Eq. (12) to obtain the solution **x**; (3) If ‖Δ**x**‖ = ‖**x**_*previous*_ − **x**‖ > *η*, then **x**_*previous*_ = **x**, go to step (1); Otherwise: go to step (4); (4) $\widehat{\mathbf{x}}=\mathbf{x}$, *β* = 2*β*, go to step (1);	
End while	
**Output** $\widehat{\mathbf{x}}$	

To find the extreme of objective function in Eq. (8), firstly the equivalent transformation ${\left\Vert \boldsymbol{\upalpha} -\boldsymbol{\Psi} \mathbf{x}\right\Vert}_2^2={\left\Vert \mathbf{G}\boldsymbol{\upalpha } -\mathbf{G}\left(\boldsymbol{\Psi} \mathbf{x}\right)\right\Vert}_F^2$ is taken; then, the coefficients in rows of **Gα** (each group) are computed separately by solving least square method. Let **α**^*i*^ = (**Gα**)_*i*, :_, (**Ψx**)^*i*^ = (**Ψx**)_*i*, :_ denote the *i*th group of **Gα** and **Ψx** respectively, we find solution by

9 $$ \underset{{\boldsymbol{\upalpha}}^i}{\min }{\left\Vert {\boldsymbol{\upalpha}}^i\right\Vert}_2+\frac{\beta }{2}\ {\left\Vert {\boldsymbol{\upalpha}}^i-{\left(\boldsymbol{\Psi} \mathbf{x}\right)}^i\right\Vert}_2^2. $$

Then, **α** can be obtained via each group computing:

10 $$ {\boldsymbol{\upalpha}}^i=\left\{\begin{array}{c}\frac{{\left\Vert {\left(\boldsymbol{\Psi} \mathbf{x}\right)}^i\right\Vert}_2-\frac{1}{\beta }}{{\left\Vert {\left(\boldsymbol{\Psi} \mathbf{x}\right)}^i\right\Vert}_2}{\left(\boldsymbol{\Psi} \mathbf{x}\right)}^i,\kern0.5em {\left\Vert {\left(\boldsymbol{\Psi} \mathbf{x}\right)}^i\right\Vert}_2>\frac{1}{\beta}\\ {}\kern1.00em 0\kern0.5em \begin{array}{cc}\begin{array}{cc}& \begin{array}{cc}&, \end{array}\end{array}& {\left\Vert {\left(\boldsymbol{\Psi} \mathbf{x}\right)}^i\right\Vert}_2\le \frac{1}{\beta\ }\end{array}\end{array}\right.. $$

2) Fix **α**, **x** can be computed by solving

11 $$ \underset{\mathbf{x}}{\min }\ \beta {\left\Vert \boldsymbol{\upalpha} -\boldsymbol{\Psi} \mathbf{x}\right\Vert}_2^2+\lambda {\left\Vert \mathbf{UFx}-\mathbf{y}\right\Vert}_2^2. $$

The minimization with respect to **x** can be solved by finding the extreme of least square problem in Eq. (11). Finally, we get

12 $$ \mathbf{x}={\mathbf{F}}^{\mathrm{H}}{\left(\beta c\mathbf{I}+\lambda {\mathbf{U}}^{\mathrm{H}}\mathbf{U}\right)}^{-1}\left(\beta {\mathbf{F}\boldsymbol{\Psi}}^{\mathrm{H}}\boldsymbol{\upalpha} +\lambda {\mathbf{U}}^{\mathrm{H}}\mathbf{y}\right), $$

where, *c* is the redundancy caused by GBRWT transform. The numerical algorithm pseudo-code is listed in Algorithm 1.

## Results

The image reconstruction was performed on a server with E5-2637 v3 (3.5G Hz) *2 CPU, 8 GB memory. The proposed method, Joint sparse reconstruction based on GBRWT (JGBRWT), is compared with the Joint sparse reconstruction method based on SIDWT (JSIDWT) [15–17], that replacing **Ψ**_g_ with SIDWT in Eq. (4) and Joint reconstruction with Bayesian Compressed Sensing (JBCS) [19], which is a state-of-the-art joint multi-contrast image reconstruction that jointly explores the gradient coefficients of multiple images. The comparison with GBRWT-based single image reconstruction [7] is also included to demonstrate the advantage of the joint reconstruction. The parameter values for JBCS are taken as the same in the cods shared by the authors (http://martinos.org/~berkin/software.html). For the proposed method and JSIDWT, *λ* is set as 10^4^.

The relative ℓ_2_ norm error (RLNE) defined as $e\left(\overset{\frown }{\mathbf{x}}\right)={\left\Vert \overset{\frown }{\mathbf{x}}-\tilde{\mathbf{x}}\right\Vert}_{\mathbf{2}}/{\left\Vert \tilde{\mathbf{x}}\right\Vert}_{\mathbf{2}}$ (in which $\tilde{\mathbf{x}}$ is ground truth and $\overset{\frown }{\mathbf{x}}$ is the reconstructed image) and mean structure similarity index measure (MSSIM) [23] served as the criteria for assessing the quality of reconstructed image quality. Smaller RLNE means lower reconstructed error and higher MSSIM indicates better structure preservation capability.

**Fig. 5:**
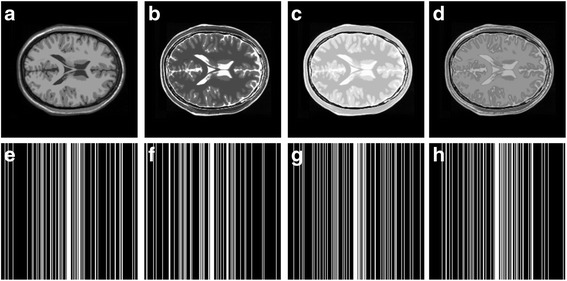
Multi-contrast Brainweb image reconstruction. **a**-**d** images with four contrasts; **e**-**h** sampling patterns operated on individual contrast images at the same sampling rate 22%

**Fig. 6:**
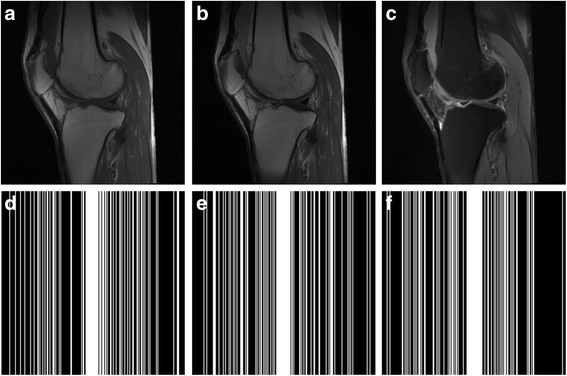
Multi-contrast knee images and under-sampling masks. **a**-**c** multi-contrast knee images with 3 kinds of contrasts; **d**-**f** 3 different sampling masks operating on each image of individual contrast with the same sampling rate 32%

**Fig. 7:**
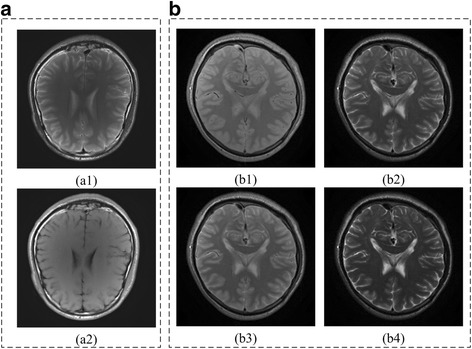
Multi-contrast brain MRI images. (A) are T2W and T1W images of one subject; (B) are images with 4 kinds of contrast for another subject by setting TE to different values

The Brainweb images (http://brainweb.bic.mni.mcgill.ca/) [24, 25] (Fig. 5) as well as the in vivo multi-contrast images were used to validate the efficiency of the proposed method. The multi-contrast knee images (Fig. 6) were acquired from GE 3 Tesla scanner (Discovery MR750W, USA) with parameters (T1W: FSE, TR/TE = 499 ms/9.63 ms; T2W: TR/TE = 2435 ms/49.98 ms, Proton density weighted image: TR/TE = 2253 ms/31.81 ms; FOV = 180 × 180 mm^2^, slice thickness = 4 mm). The multi-contrast brain images (Fig. 7) were acquired from SIMENS 3 Tesla scanner (MAGNETOM Trio Tim, Germany) with parameters (T2W: TSE, TR/TE = 3000 ms/66 ms,; T1W, FLAIR: TR/TE = 3900 ms/9.3 ms,; FOV = 200 × 200 mm^2^, slice thickness = 5 mm) for Fig. 7(A) and TSE: TR = 4000 ms, FOV = 192 × 192 mm^2^, slice thickness = 3 mm, ∆TE =8 ms for the multi-echo data in Fig. 7(B).

**Fig. 8:**
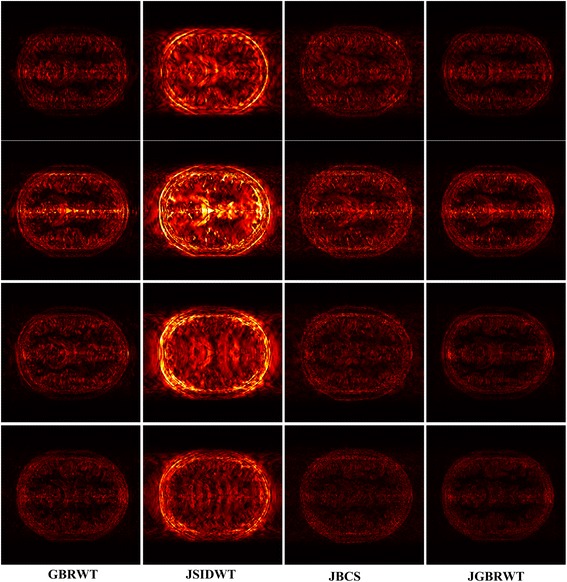
Brainweb reconstruction errors (× 5) with 22% sampled data (Cartesian). Rows 1–4 correspond to multi-contrast images shown in Fig. 5 (a-d) respectively. Columns 1–4 denote the results obtained from individual reconstruction based on GBRWT, JSIDWT, JBCS and JGBRWT reconstruction respectively

**Fig. 9:**
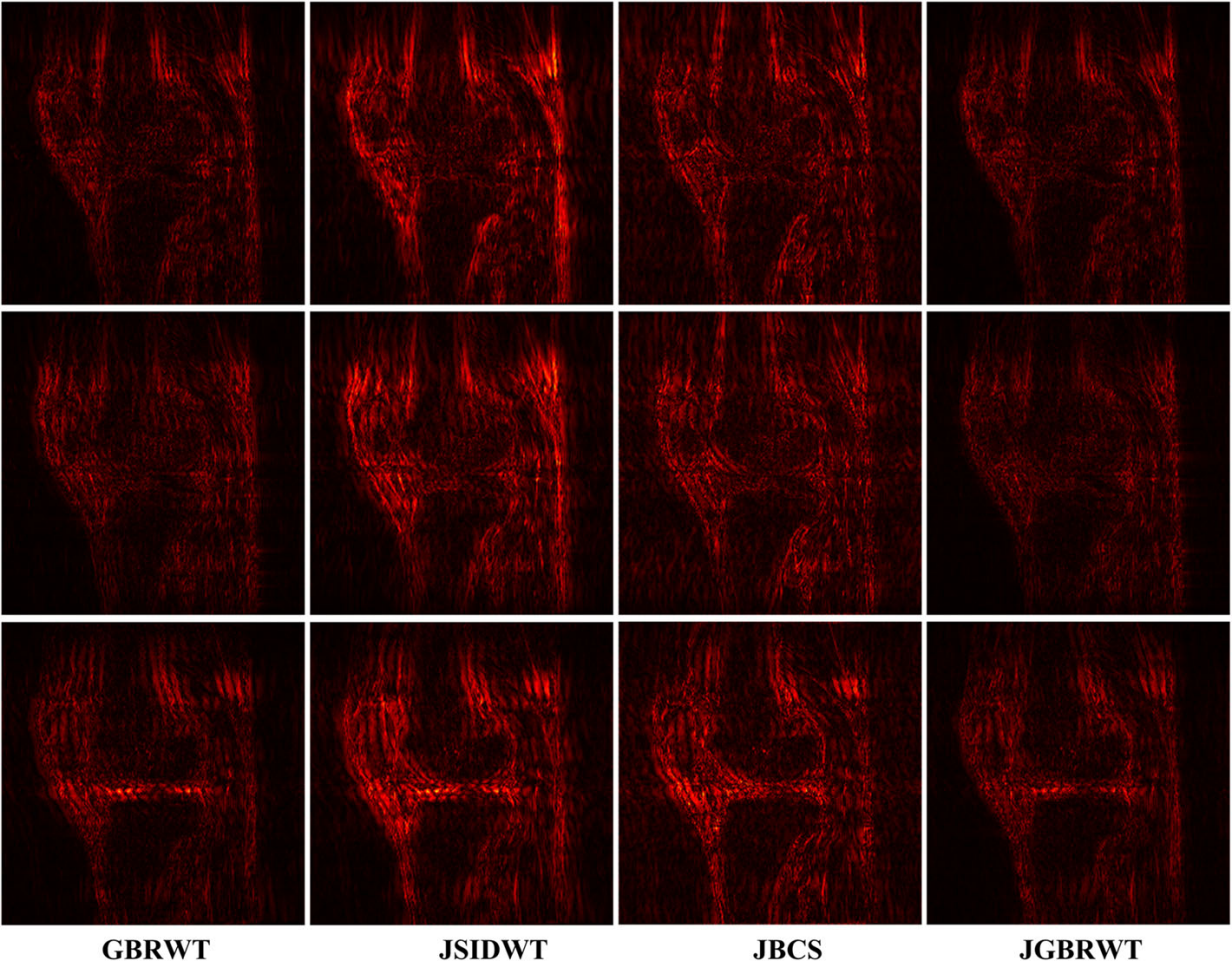
Reconstruction errors of knee images (_× 5_) with 32% sampled data (Cartesian). Rows 1–3 correspond to multi-contrast images shown in Fig. 6(a-c) respectively. Column 1–4 denote the results obtained from GBRWT, JSIDWT, JBCS and JGBRWT reconstruction respectively

**Fig. 10:**
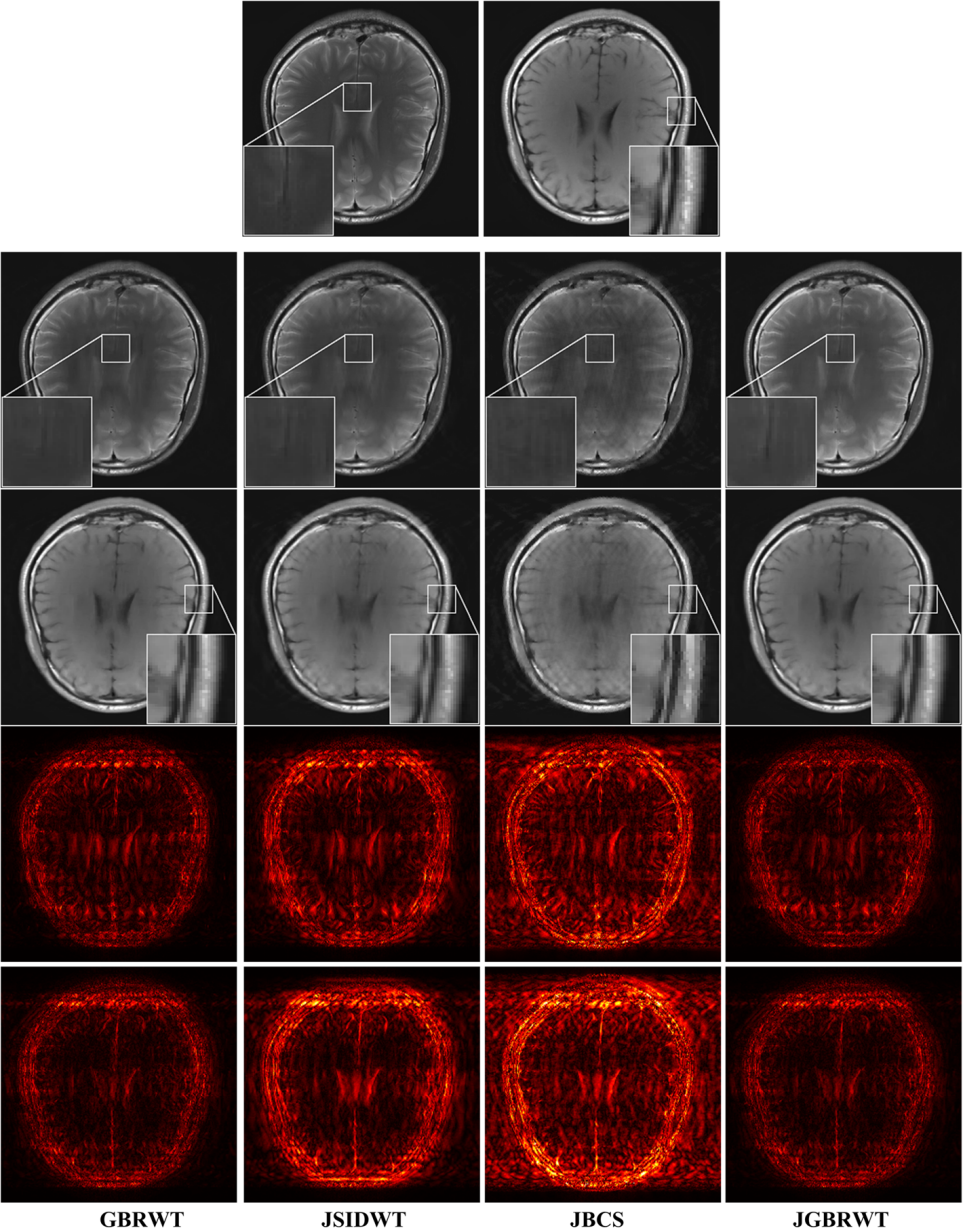
Reconstructed brain images and corresponding errors (_× 5_) with 27% sampled data. The 1st row shows fully sampled multi-contrast images, the 2nd and 3rd rows are reconstructed images and last two rows are reconstruction errors corresponding to the 2nd and 3rd rows, respectively. Columns 1 to 4 denote the results obtained from GBRWT, JSIDWT, JBCS and JGBRWT, respectively

Fully sampled multi-contrast MRI images shown in Figs 5, 6 and 7 are used for the experiment of under-sampling and joint sparse reconstruction. Reconstruction errors shown in Figs. 8, 9 and 10 reveal that the proposed method outperforms the JSIDWT and JBCS. The lower error of the proposed method indicates better fidelity and edge-preserving capabilities compared with JSIDWT and JBCS. Besides, the reconstruction errors were reduced when comparing the proposed method with single image reconstruction based on the same GBRWT transform, implying that the improvement obtained by joint reconstruction over single image reconstruction.

Tables 1, 2 and 3 show RLNEs and MSSIMs of reconstructed images. These criteria indicate that the proposed method gained the highest MSSIM and the lowest RLNE, and thus recovered the images most faithfully.

**Table 1:** RLNE/MSSIM of Brainweb images with 22% Cartesian sampled data in Fig. 5

Images	GBRWT	JSIDWT	JBCS	JGBRWT
Fig. 5(a)	0.0688/0.9321	0.1643/0.6944	0.0985/0.7091	0.0365/0.9799
Fig. 5(b)	0.1048/0.9206	0.2284/0.6774	0.1336/0.7143	0.0609/0.9701
Fig. 5(c)	0.0492/0.9038	0.1233/0.6742	0.0525/0.7665	0.0232/0.9815
Fig. 5(d)	0.0660/0.9100	0.1222/0.7265	0.0596/0.8005	0.0306/0.9821

**Table 2:** RLNE/MSSIM of knee images with 32% Cartesian sampled data in Fig. 6

Images	GBRWT	JSIDWT	JBCS	JGBRWT
Fig. 6(a)	0.0615/0.9560	0.0932/0.9211	0.0850/0.9064	0.0582/0.9624
Fig. 6(b)	0.0629/0.9605	0.0976/0.9234	0.0890/0.9004	0.0534/0.9704
Fig. 6(c)	0.0883/0.9337	0.1216/0.9312	0.1238/0.9180	0.0823/0.9607

**Table 3:** RLNE/MSSIM of reconstructed brain images with 27% sampled data in Fig. 7

Images	GBRWT	JSIDWT	JBCS	JGBRWT
Fig. 7(a1)	0.0505/0.9612	0.0924/0.9089	0.1297/0.7759	0.0487/0.9636
Fig. 7(a2)	0.0762/0.9593	0.1103/0.9149	0.1502/0.7817	0.0733/0.9639

One typical brain image reconstruction with 27% sampled data are shown in Fig. 10. In the zoomed-in area of the 2nd row, the sulcus of the T2W image appears in the middle of the fully sampled and JGBRWT reconstructed images, but nearly disappears for JBCS reconstruction. In the marked region of the 3rd, the proposed method leads to more consistent reconstruction with the fully sampled image than other methods. These improvements are also confirmed by the error images in the last two rows.

### 2D under-sampling

**Fig. 11:**
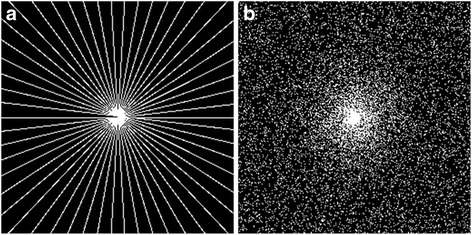
The 2D under-sampling mask. **a** is pseudo-radial sampling with sampling rate 11%, **b** is random sampling with sampling rate 15%

The 2Dunder-sampling patterns (Fig. 11) was explored to demonstrate the potential applications of the proposed method in 3D imaging, in which 2D phase encoding plane can be under-sampled.

**Fig. 12:**
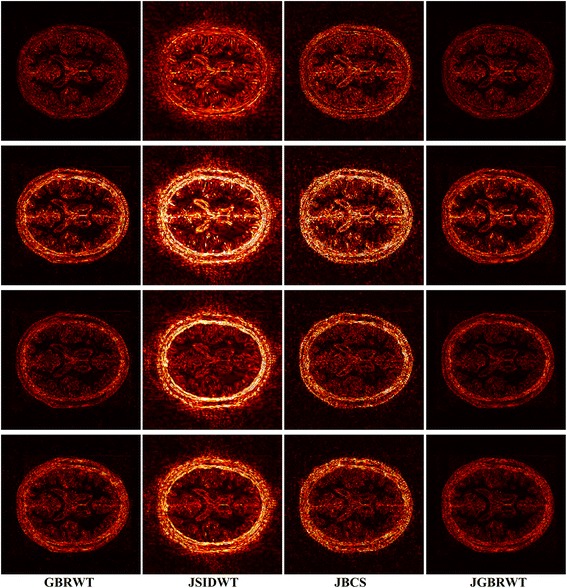
Reconstructed error (_× 5_) using 2D pseudo-radial under-sampling with 11% sampled data of Fig. 5. Rows 1–4 correspond to multi-contrast images shown in Fig. 5 (a-d) respectively. Columns 1–4 denote the results obtained from GBRWT, JSIDWT, JBCS and JGBRWT reconstruction respectively

**Fig. 13:**
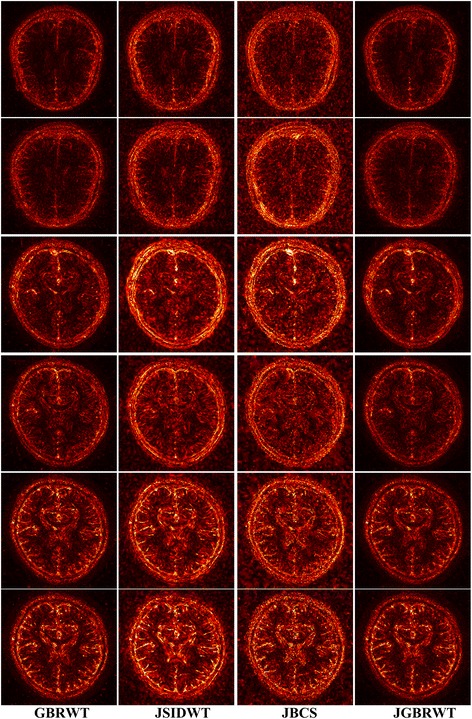
Reconstructed error (_× 5_) using 2D Random under-sampling, with 15% sampled data in Fig. 7(a1-a2), and 26% sampled data in Fig. 7(b1-b4). Rows 1–6 are reconstruction errors correspond to multi-contrast images shown in Figs. 7(a1-a2, b1-b4) respectively. Columns 1–4 denote the results obtained from GBRWT, JSIDWT, JBCS and JGBRWT respectively

Brainweb reconstructed errors shown in Fig. 12 demonstrate that on the simulated database, the lowest reconstruction errors were obtained with the proposed method. The corresponding RLNE/MSSIM are shown in Table 4. Figure 13 implies that the proposed method led to the lowest brightness in the error images and thus maintained fidelity best. The criteria listed in Tables 4 and 5 indicate that the proposed method achieved the highest MSSIM and the lowest RLNE on the tested dataset.

**Table 4:** RLNE/MSSIM for the reconstruction using 11% pseudo-radial k-space data of Fig. 5

Images	GBRWT	JSIDWT	JBCS	JGBRWT
Fig. 5(a)	0.0443/0.9779	0.1011/0.6966	0.0835/0.8595	0.0395/0.9813
Fig. 5(b)	0.1036/0.9510	0.2118/0.6246	0.1632/0.7865	0.0811/0.9680
Fig. 5(c)	0.0327/0.9681	0.1008/0.6666	0.0663/0.8720	0.0304/0.9789
Fig. 5(d)	0.0606/0.9476	0.1206/0.7062	0.0890/0.8745	0.0444/0.9757

**Table 5:** RLNE/MSSIM for the reconstruction using 15% randomly sampled k-space data of Fig. 7(A) and Fig. 7(B)

Images	GBRWT	JSIDWT	JBCS	JGBRWT
Fig. 7(a1)	0.0537/0.9526	0.0735/0.9222	0.0973/0.8220	0.0515/0.9562
Fig. 7(a2)	0.0735/0.9563	0.0957/0.9231	0.1097/0.8480	0.0722/0.9589
Fig. 7(b1)	0.0844/0.8820	0.1315/0.7739	0.1366/0.7129	0.0820/0.8955
Fig. 7(b2)	0.0935/0.8883	0.1278/0.8065	0.1302/0.7698	0.0817/0.9142
Fig. 7(b3)	0.1327/0.8611	0.1691/0.7821	0.1707/0.7411	0.1184/0.8876
Fig. 7(b4)	0.1624/0.8481	0.2158/0.7544	0.2054/0.7602	0.1558/0.8688

### Different sampling rates

**Fig. 14:**
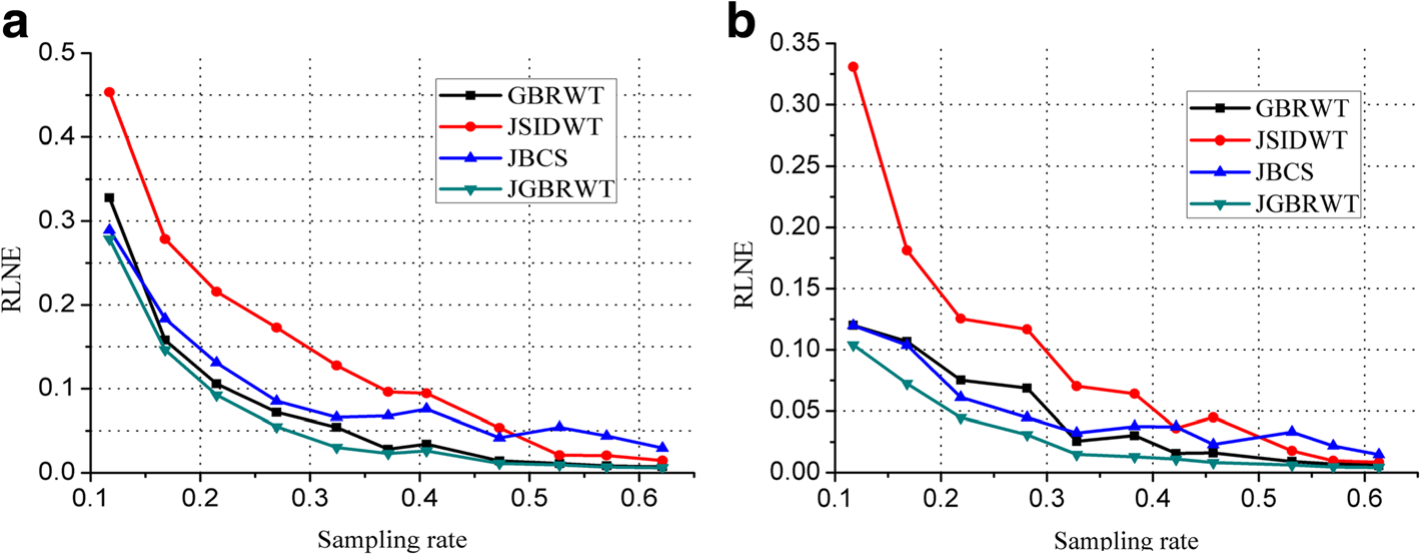
RLNEs evaluation at various sampling rates. **a** and **b** display the RLNEs with regard to the fully sampled data shown in Figs. 5(b) and (d) under Cartesian under-samplings

The curves in Fig. 14 show that the RLNEs decreased with sampling rate increased. The RLNE line of the proposed JGBRWT method (dark green line) is lower than that of GBRWT (or contrast-by-contrast reconstruction, black line) with the same GBRWT representation, indicating benefits are achieved by utilizing joint sparsity among multi-contrast images. The JGBRWT also outperforms other joint reconstruction method, including JSIDWT (red line) and JBCS (blue line), in terms of lower RLNEs at all sampling rates.

### The same sampling patterns

The proposed method is compatible to same or different sampling patterns. Reconstruction criteria in Table 6 show that the proposed method outperforms the compared ones under the same sampling patterns. Besides, at the same sampling rate, using different sampling patterns lead to better evaluation criteria than using same sampling patterns (Table 6 vs. Table 1).

**Table 6:** RLNE/MSSIMs under same sampling patterns (sampling rate 22%)

Images	GBRWT	JSIDWT	JBCS	JGBRWT
Fig. 5(a)	0.0705/0.9305	0.1683/0.6858	0.1019/0.7422	0.0420/0.9760
Fig. 5(b)	0.0986/0.9322	0.2308/0.6536	0.1362/0.7371	0.0696/0.9661
Fig. 5(c)	0.0412/0.9309	0.1340/0.6505	0.0540/0.7768	0.0248/0.9788
Fig. 5(d)	0.0563/0.9366	0.1382/0.7036	0.0635/0.8190	0.0331/0.9796

## Discussions

### Limitations on choosing image to train the graph

**Fig. 15:**
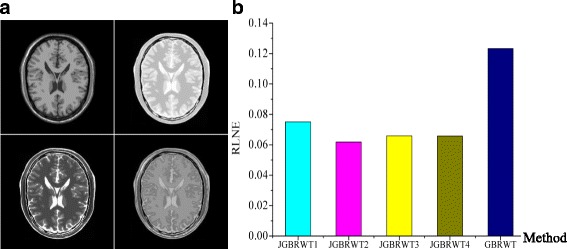
Effect of reference images. **a** multi-contrast images (contrast 1–4 are arranged from left to right, from top to bottom); **b** average reconstruction RLNEs of multi-contrast images in JGBRWT using different references and in GBRWT. Note: JGBRWT1 denote the JGBRWT using image of contrast 1 as reference, and so on

Choose an arbitrary pre-reconstructed image as reference will lead to reconstruction errors (RLNEs) slightly change as shown in Fig. 15. But the RLNEs are still much lower than single image reconstruction. A possible way in the future work is to train a GBRWT jointly from all the under-sampled multi-contrast images to make full use of the common/complementary information of multi-contrast images.

### Limitations on un-registered images

**Fig. 16:**
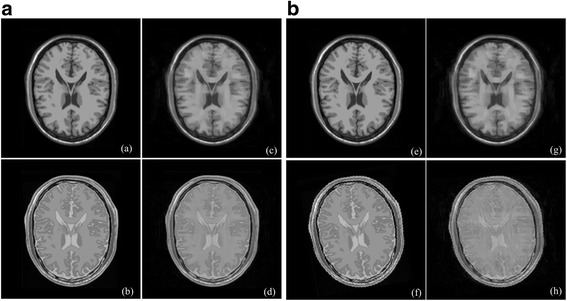
Joint sparse reconstruction with aligned and misaligned multi-contrast images at sampling rate 16%. (**a**)-(**b**) are aligned fully sampled images; (**c**)-(**d**) are reconstruction with RLNE = 0.1016 and RLNE = 0.0667, respectively; (**e**)-(**f**) are misaligned fully sampled images; (**c**)-(**d**) are reconstruction with RLNE = 0.1277 and RLNE = 0.1071, respectively

Un-registered multi-contrast images will go against the joint sparsity assumption, and thus affect joint reconstruction performance. Reconstructed images of aligned and misaligned multi-contrast images (we simulate misalignment by rotating Fig. 16(a) with 10 degrees) shown in Fig. 16 demonstrate that misalignment will make the detail reconstruction deteriorated. RLNE obviously increased in sparse reconstruction of misaligned multi-contrast images. Improved image reconstruction is expected by incorporating the registration into image reconstruction process as it was done in [6], which would be interesting as a future work.

### Computation complexity

The main step of numerical algorithm to solve the proposed joint reconstruction problem include a soft thresholding to solve **α** and a one-step computation to solve **x**, which is with the same computation complexity as single contrast image reconstruction, but with more data to compute, and thus no obvious additional computational burden.

Program at our platform (E5–2637 v3 (3.5G Hz) *2 CPU, 8 GB memory) shows that, the SIDWT-based single image reconstruction need 20 s, and SIDWT-based joint reconstruction need 100 with 4 different contrast images at low sampling rate. The GBRWT-based single image reconstruction need 200 s and GBRWT-based joint reconstruction need 103 s with 4 different contrast images at low sampling rate.

### Experiment with noise

**Fig. 17:**
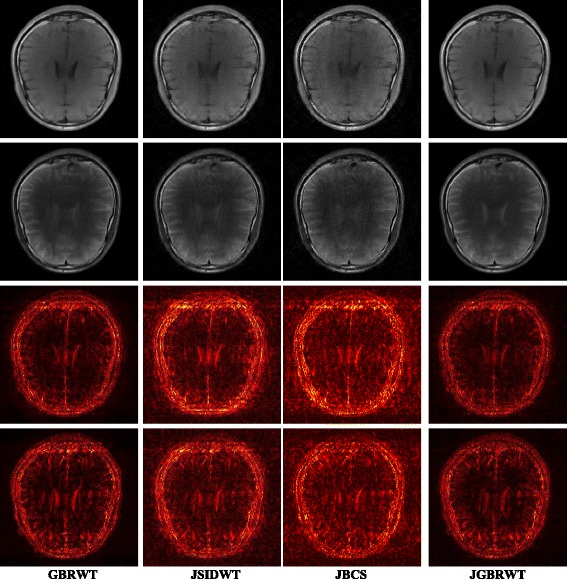
Noisy experiment (with 27% sampled data) under Gaussian white noise with variance *σ*^2^ = 0.02. Rows 1–2: reconstructed images; Rows 3–4: magnitude errors (× 5)

Multi-contrast images in Fig. 7(A) in the manuscript are used in noise experiment. Noisy data are simulated by adding Gaussian white noise with variance *σ*^2^ = 0.02 on real and imaginary part of k-space data. Figure 17 demonstrate that the proposed method outperforms the compared ones in preserving image structures as well as removing noise. According to Table 7 the proposed method achieves lowest RLNEs, highest MSSIMs and highest SNRs. The signal to noise rate (SNR) is defined as *SNR* = 10log_10_(*μ*/*σ*), where u is the mean of image density and δ is the standard deviation of the noise extracted from the image background.

**Table 7:** SNR/MSSIM/RLNEs in noise experiment with 27% sampled data

Images	GBRWT	JSIDWT	JBCS	JGBRWT
Fig. 7(a1)	18.59/0.9108/0.0814	15.51/0.7977/0.1161	14.28/0.7168/0.1337	19.58/0.9247/0.0726
Fig. 7(a2)	15.74/0.9031/0.1179	14.10/0.7845/0.1424	12.94/0.7012/0.1627	16.61/0.8241/0.1067

### Parameters

**Fig. 18:**
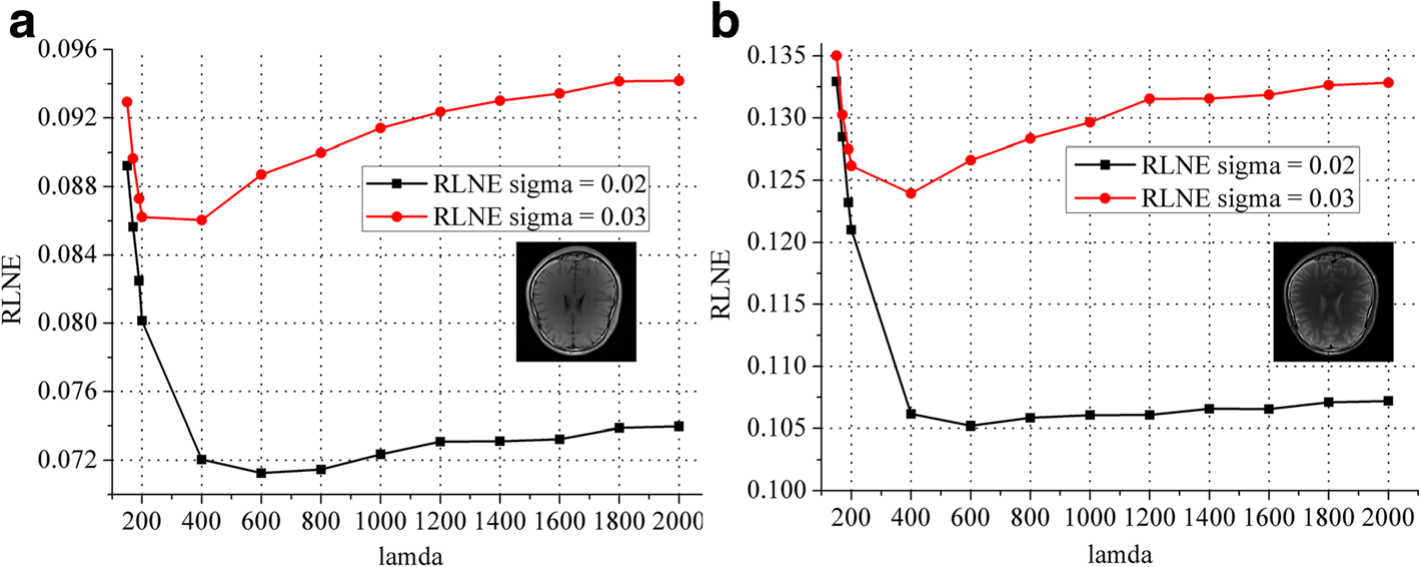
The optimal *λ* under different noise levels. **a**-**b** are RLNE curves for each contrast image. Note: Two contrast images under 1D under-sampling pattern at sampling rate 27% are reconstructed using the proposed JGBRWT

Two noise level are considered (Gaussian white noise with variance *σ*^2^ = 0.02 and *σ*^2^ = 0.03) in testing *λ*. The optimal *λ* for *σ*^2^ = 0.02 and *σ*^2^ = 0.03 are 600 and 400 respectively on the tested data according the curve shown in Fig. 18.

The parameters of GBRWT include patch-size and decomposition levels, which have been discussed in [7]. The suggested patch-size in GBRWT are from 4 × 4 to 7 × 7, and suggested decomposition level is 3–5 level. We use the patch-size 7 × 7 and do 5 level decomposition in this experiment.

## Conclusions

A new approach is proposed to simultaneously explore the adaptive sparse image representation under graph-based redundant wavelet transform and the joint sparse reconstruction of multi-contrast MRI images. Experimental results in synthetic and in vivo MRI data demonstrate that the proposed method can achieve lower reconstruction errors than the compared methods. With this high quality image reconstruction method, it is possible to achieve the high acceleration factors by exploring the complementary information provided by multi-contrast MRI.

## Abbreviations

ADMC: Alternating direction method with continuity
GBRWT: Graph-based redundant wavelet transform
JBCS: Joint reconstruction based on Bayesian compressed sensing
JGBRWT: Joint sparse reconstruction based on GBRWT
JSIDWT: Joint sparse reconstruction based on SIDWT
MRI: Magnetic resonance imaging
MSSIM: Mean structure similarity index measure
PDW: Proton density weighted MRI image
RLNE: Relative ℓ_2_ norm error
SIDWT: Shift invariant discrete wavelet transform
T1W: T1 weighted MRI image
T2W: T2 weighted MRI image
